# A national survey of the prevalence of schistosomiasis and soil transmitted helminths in Malaŵi

**DOI:** 10.1186/1471-2334-4-49

**Published:** 2004-11-16

**Authors:** Cameron Bowie, Bernadette Purcell, Bina Shaba, Peter Makaula, Maria Perez

**Affiliations:** 1 Department of Community Health, College of Medicine, University of Malawi, Blantyre, Malawi; 2 Sussex Health Protection Unit, Lewes, East Sussex, UK; 3 Community Health Sciences Unit, Ministry of Health, Lilongwe, Malawi; 4 PO Box 345, Mangochi, Malawi; 5 Department of Microbiology, College of Medicine, University of Malawi, Blantyre, Malawi

## Abstract

**Background:**

Past estimates have put the prevalence of schistosomiasis between 40% and 50% in the Malawi population overall based on studies undertaken ten years or more ago. More recent surveys in known high risk areas find similar levels. However control measures, changing ecology and migration may have led to changes in the prevalence of schistosomiasis in different parts of Malawi. A national schistosomiasis and soil-transmitted helminth (STH) survey was undertaken to measure the distribution, prevalence and intensity of infection in November 2002.

**Methods:**

A school was selected randomly from a random sample of 30 Traditional Authorities stratified by six distinct ecological zones, and 1,664 year 3 pupils (9–10 year olds) were questioned about recent illnesses and "red urine". Samples of urine and faeces were examined for the presence of eggs using the standard Kato-Katz technique for soil-transmitted helminths and intestinal schistosomiasis and urine samples using the filtration technique for *Schistosoma haematobium*.

**Results:**

The prevalence of *Schistosoma mansoni *is 0.4% (95% CI 0–1.3%), *S. haematobium *6.9% (95% CI 1.9 – 11.9%), hookworm 1.3% (95% CI 0.4–2.3%), Ascariasis 0.5% (95% CI 0.1–1.0%) and trichuriasis 0% in year 3 pupils (modal age 10 years of age). Intensity of infection is low for all infections except for 2.5% who have high intensity *S. haematobium *infection. The "red urine" question is 67% sensitive and 80% specific for positive *S. haematobium *microscopy.

**Conclusions:**

The reduction in prevalences may be real as a result of recent control measures, or false if historical results were based on surveys of high risk populations. Another explanation is that this survey used an unrepresentative sample of schools. Detailed analysis suggests this is unlikely.

Recommendations include the use of a 30% positive threshold for the "red urine" screening question to be used in schoolchildren in high prevalence areas.

This survey, based on a national probability sample excluding the northern region lakeside area, finds much lower overall prevalence and intensity of schistosomiasis and STHs than previous estimates based on selected surveys. Disease control featuring chemotherapy may be having a profound effect. The localised nature of the distribution of the infections means that control programmes may work best if undertaken at district level or below. "Red urine" questionnaire surveys may help identify hot spots.

## Background

Chronic infections with soil-transmitted helminths and schistosomiasis are common in Malaŵi and cause considerable morbidity [1]. The Ministry of Health wishes to establish the distribution of these infections prior to a reassessment of national policy regarding their control.

All water bodies in Malawi are considered to be potential transmission sites for schistosomiasis [2]. The most recent national estimate puts the prevalence of schistosomiasis between 40% and 50% of the population [3]. This is based on surveys carried out prior to 1991. Numerous local surveys have been conducted more recently in different parts of the country [4-7]. In a survey carried out by Randall et al in 1996 in northern Malawi, *S. mansoni *and *S. haematobium *were detected in 27% and 20% of schoolchildren respectively [8]. A survey performed by the Bilharzia Control Programme in Mangochi reported in 1999 showed between 60–80% of school children living along the lakeshore has *S. haematobium *[9]. This level of infection was confirmed in a later survey performed by the Lakeshore Schistosomiasis Control Project, which found the prevalence amongst school aged children (5–15 years old) to be as high as 80% along the lake shore, with some areas having 100% infection rates [2].

In Malawi there is little contemporary information available on helminth infection. Furthermore, fragmented control measures, changing ecology [10] (such as considerable water resource development and deforestation in the last 10 years) and migration may have led to marked changes in the prevalence of schistosomiasis within regions in Malawi since previous studies were done.

A national schistosomiasis and soil-transmitted helminth (STH) survey was undertaken to measure the distribution, prevalence and intensity of infection in November 2002. Results from the survey are reported in this paper.

## Methods

Methods follow recent WHO advice to allow comparison with other international studies [11]. This includes a symptom and illness questionnaire and urine and faecal sampling. The use of questionnaires as a rapid assessment tool for urinary schistosomiasis has been evaluated in multi-country studies [4].

**Figure 1:**
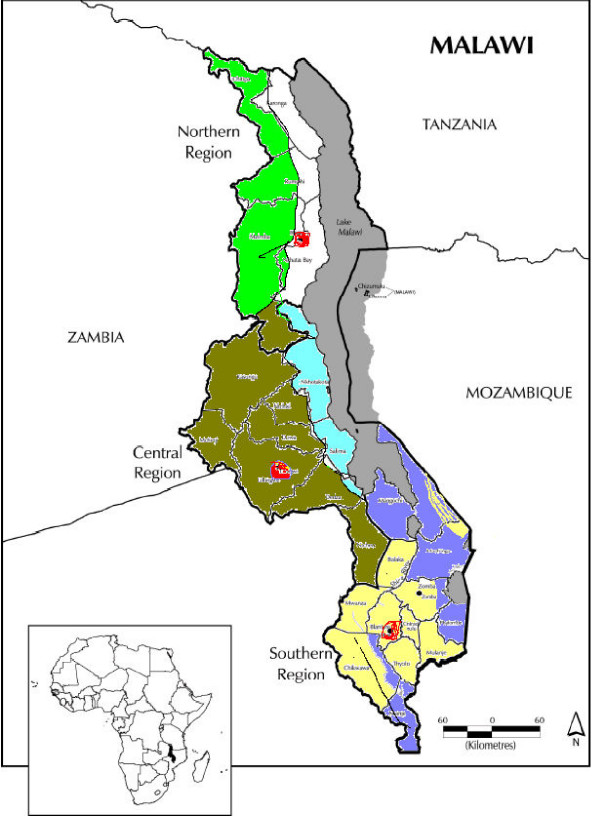
Seven distinct ecological zones in Malaŵi

Seven areas (with the number of Traditional Authorities in each) represent the seven distinct ecology and soil conditions in Malaŵi (figure 1):-

Northern Region highland (23)

Northern Region lakeside

Central Region highland (62)

Central Region lakeside (17)

Southern Region highland (51)

Southern Region lakeside (31)

Urban (3)

The northern region lakeside had recently been surveyed by the Karonga Prevention Study [8]. To save expense this ecological area was not resurveyed. Hence only six areas were included in this survey.

### Study population

Primary-school children were chosen as the target population. There are several reasons for conducting the survey in this age group. Children consistently have the highest prevalence and transmission of schistosomiasis and soil-transmitted nematodes (except hookworm); treatment via schools on a national scale is feasible [12] (and primary school enrolment is reasonably high in Malawi) and extremely cost-effective [13]. Third-year school classes (9–10 year olds) are commonly surveyed and this was the age-group for this survey.

### Sample frame and selection

**Figure 2:**
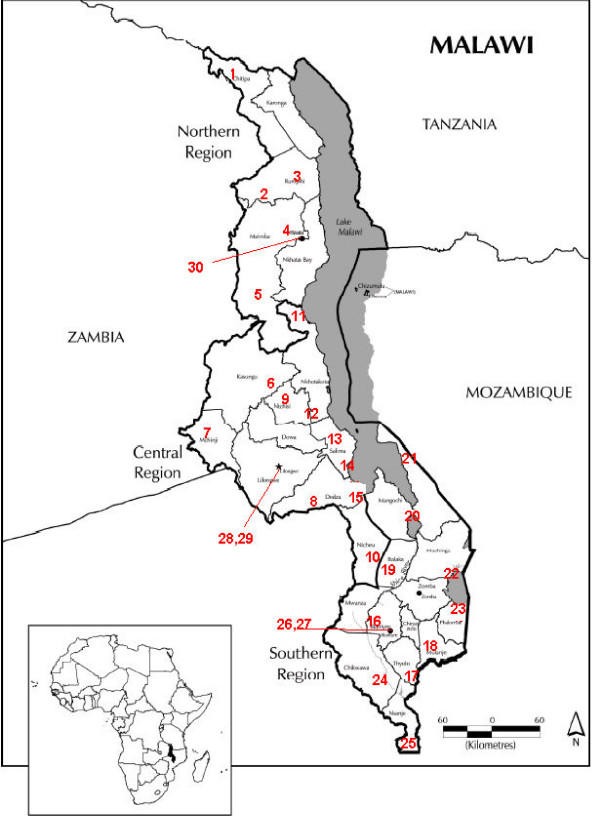
Map of Malawi with selected schools

The sample was selected using a stratified multi-stage cluster design [14]. The six ecological zones constituted the strata. School children were clustered within schools which were clustered within Traditional Authorities (TA). Five Traditional Authorities (TA) were randomly selected from the total in each ecological zone using a computer generated random numbers technique. A list of all primary schools in these TAs was compiled by contacting the relevant District Education Officer and one school was selected at random. Private schools were included though the vast majority were government schools. In the three urban districts of Blantyre, Lilongwe and Mzuzu five schools were selected. A total of 30 schools were surveyed between 25 September and 14th November 2002. A census of all children attending standard 3 who were present on the day of the survey was taken and all were enrolled. Thus between 36 and 71 third year schoolchildren (aged 8–10 years) in 5 schools selected at random from each of six ecologically distinct regions of the country were sampled (Figure 2). A total of 1664 children were enrolled.

### Questionnaire

All children were administered a standard symptom questionnaire by a class teacher in their local language [see Additional file 1]. The questionnaire was completed before specimen collection and was designed to assess general health without biasing responses towards helminthiasis in particular. Children were asked about symptoms during the past month [e.g. cough, itch, headache, fever] and whether they had particular illnesses during the past month [e.g. malaria, diarrhoea, skin, eye disease, bilharzia].

### Specimen collection methods

School children were asked to provide a stool sample and at least 10 ml of urine (collected mid-morning). Stool samples were stored in a cool box during transfer to the laboratory, and then kept at 4°C until processed within 24 hrs of collection.

### Urine and faecal analysis

Stool samples were tested for the presence of eggs using the standard Kato-Katz technique [15] for soil-transmitted helminths and intestinal schistosomiasis. Samples with ascaris more than 50,000 eggs per gram (epg), hookworms more than 4,000 epg, trichuria more than 10,000 epg and *S. mansoni *more than 400 epg were defined as high intensity. Urine samples were tested using the filtration technique for *S. haematobium*. Infection with *S. haematobium *was classified as high intensity if at least 50 eggs per 10 ml of urine were detected or there was visible haematuria [16].

### Data analysis

Data were double entered into EpiInfo and then exported into STATA v7 for initial analysis. SPSS was used for chi squared, paired-t and McNemar tests. EpiInfo 2002 was used to calculate adjusted prevalence rates for ecological zones and national prevalence. The prevalences and 95% confidence intervals were computed incorporating the stratification by ecological zones. Sampling weights were computed as the product of the number of TA's in an ecological zone times the number of schools in the selected TA. For the urban zone, weights were adjusted for the fact that there were only three TA's and two schools were selected from two of them (Complex sample frequency analysis, Epi Info 2002, CDC Atlanta). The relationship between the prevalence and intensity of *S. haematobium *infection in schools was investigated using Pearson product moment correlation coefficient (SPSS).

## Results

### The sample

A total of 1,664 children were surveyed. Between 36 and 71 children were included from each school. There were 1,662 completed questionnaires, 1,546 stool samples and 1,638 urine specimens collected. Just over half (50.7%) of the sample was female. The mean age (for 1,658 children where age was recorded) was 10.7 years [range 6–17 years]. The mean age of boys was 10.9 years and of girls 10.5 years.

### Prevalence and intensity of infection

#### Schistosomiasis

A total of 140 children from 19 different schools had *S. haematobium *detected by urine microscopy, of which 45 had high intensity infection. The prevalence in schools ranged between 0 (11 schools) and 43.1% (a school in the southern lowlands). Twenty one children from eight different schools had visible haematuria. Only 8 children from 2 different schools had *S. mansoni *detected by stool microscopy and none were high intensity infections.

Overall, the national prevalence of *S. mansoni *was found to be 0.4%. The national prevalence of *S. haematobium *was found to be 6.9% (95% CI 1.9–11.9%) (Table 1). 2.5% of these were high intensity infections [range 0–23.3%]. There was a clear association between prevalence and intensity (r = 0.9, n = 30, p = <0.01); schools with high prevalence of *S. haematobium *also had high intensity of infection.

**Figure 3:**
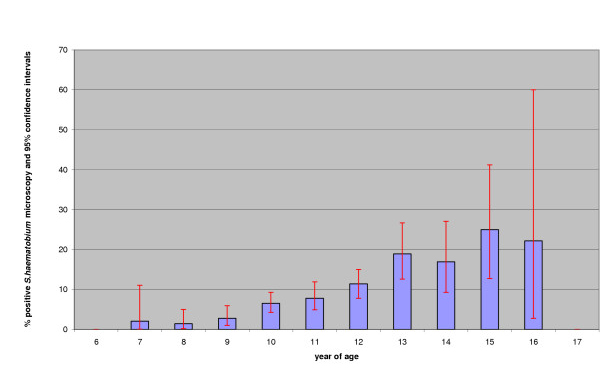
Prevalence of *S. haematobium *in Standard 3 schoolchildren by year of age

There was a wide range of ages in standard 3, with mode of 10 years of age. The observed prevalence was higher in older children (see figure 3) and boys (9.0%) compared with girls (8.2%).

#### Soil transmitted helminths

The number of children with detectable helminth infestation was low. Twenty-six children from 13 schools had hookworm detected from stool, none of which were high intensity. Eighteen children from nine different schools had ascaris eggs detected and no trichuris eggs were detected at all. There was no obvious pattern of co-infection with schistosomiasis and soil-transmitted helminths across the schools sampled; in 16 schools there was concordance with either both or neither type of infection detected; in 14 schools there was no concordance.

The prevalence of STHs was much lower than that of schistosomiasis (Table 1). Only 1.8% [95% C I 0.6–3.1%] of children had evidence of infection with any of hookworm, ascaris or trichuriasis on stool microscopy. There were no heavy intensity STH infestations detected.

### Prevalence and intensity of infection by ecological region

#### Schistosomiasis

*S. haematobium *infection was detected in all ecological areas (Table 2). The highest rates were in the Southern Lowland (23.2%) and the Central lowland (9.5%); the other four areas examined all showed a prevalence of between 2% and 7.4%. There was a wide range in the prevalence of *S. haematobium *infection in all areas. *S. mansoni *was rarely detected, and only found in the Southern Highlands (1.3%).

To assess the sampling method used to select schools we classified the selected schools into high and low expected prevalence of *S. haematobium *based on ecology and known previous prevalence and compared our survey results with these expectations. Of the 12 expected high prevalence schools seven were found to be high and 5 unexpectedly low. Of the 18 expected low prevalence schools 12 were found to be low and 6 unexpectedly high. Unexpected results were found in the expected low prevalence as much as in the expected high prevalence schools using the McNemar test for symmetry actual (p = 0.29).

We also compared the historical results for Malawi districts compiled by the London School of Hygiene and Tropical Medicine for the Schistosomiasis Control Initiative in 2001 (personal communication with Simon Brooker) with our results. Prevalence has fallen overall by 24% (95% confidence intervals of 16–32%). A formal paired t-test finds this reduction is true for low as well as high prevalence schools (t = 6.4 with 29 degrees of freedom and highly statistically significant).

### Soil transmitted helminths

There was also a low prevalence of STHs in all areas, though urban areas had the highest rates for any STH (6.6%) and hookworm was most prevalent in the Northern Highland area (2.6%). There were no STHs detected in the Central lakeshore schools.

### Questionnaire responses

Total number of respondents was 1,662 (Table 3). It is possible to calculate the sensitivity and specificity of the "red urine" question in respect of the microscopic presence of *S. haematobium *(Table 4). The sensitivity of the "red urine" question in respect of *S. haematobium *being seen by microscopy is 67% and the specificity 80%. At a national average prevalence of 8.5% the "red urine" question has a positive predictive value of 24% and a negative predictive value of 96%. At the higher prevalence (22.5%) found in the Southern Lowlands zone the positive predictive value is 49% and the negative predictive value is 89%.

## Discussion

### Prevalence and distribution

The overall prevalence of schistosomiasis is well below expectations. Based on previous studies in Malawi the overall prevalence of schistosomiasis was thought to be between 40% and 50% in the population. Of course these were surveys of selected populations, perhaps undertaken in the season of high transmission. Certainly the disease is localised in Malawi. In the study conducted in the northern lakeshore area in 2000 by Randall et al [8], schoolchildren from four schools were screened and there was a wide range of prevalence: 5%–57% on *S. haematobium *and 6%–42% in the case of *S. mansoni*. The school located closest to rice fields had a high prevalence of schistosomiasis. In another recent survey carried out at the Cape McClear peninsula in the Southern Lake zone results showed markedly different *S. haematobium *and *S. mansoni *prevalence amongst adjacent villages situated on the lake shore and inland (Paul Bloch, personal communication).

Our survey, representative of all schoolchildren in the country, and undertaken just before the rainy season, suggests far lower levels of 7% for *S. haematobium *and 0.4% for *S. mansoni*. The finding highlights two important features of schistosomiasis in Malawi. Firstly, these infections are highly localised and secondly they are not common in general. As expected higher prevalence rates are found in the Southern lake/lowlands zone. *S. mansoni *does not seem to be a generalised problem in any particular ecological area in the country.

The overall prevalence of STHs is lower than expected. The survey conducted in Karonga District (Randall et al., 2000 [8]) finds a higher prevalence of STHs as well as of *S. mansoni *and *S. haematobium*. The differences could be due to seasonal variation (the survey in Karonga district was conducted after the rainy season). This possibility is supported by a low prevalence found in Karonga district in samples collected in November 2001, prior to the rains (unpublished observation, MA Perez). Supporting evidence is seen in the prevalence rates found in urban areas where rains would not affect transmission as much as in rural areas. Similar results are found in previous studies in urban Blantyre: *S. mansoni *(0%), Ascaris (18%) and Hookworm (0.4%) [7]. Another explanation for the higher prevalence rates found in Karonga District could be the different methodology used to process the stools. The Kato-Katz method [16] was used during our survey in order to calculate intensity of infection. Due to time and personnel constraints, only one Kato-Katz slide was read from each sample, and this could have affected the sensitivity of our testing [17,18]. On the other hand the survey in Karonga District was done using a commercial kit (Parasep^®^, Intercep Ltd) in order to concentrate the stools and achieve high diagnostic sensitivity. Because of the dissimilar laboratory methods we have been unable to include results for the Northern Lakeland Ecological zone in the estimate of overall prevalences for the whole country. The northern lakeshore zone has tended to have high prevalences. The impact of omitting this zone is likely to underestimate the national results. We intend to survey the zone using our own laboratory methods when funds are available to complete the national picture.

Although this survey is designed to be representative of this age group in Malawi, it is limited in scale by the budget ($12,000). This is an important limitation when one considers that the distribution of schistosomiasis in populations is likely to be highly localized [19]. The population sampled is representative of the general population of school children in standard three with respect to age and sex distribution. However, this survey does not represent children in Malawi who do not attend school or who were absent on the day of the survey due to illness, or who live in the northern lakeland zone. Despite the introduction of free primary education, the net enrolment rate in Malawi is 78%. There is little difference between poor and non-poor households in regard to the proportion of primary school-aged children in school [20]. However, poor children are likely to drop out before reaching standard 5, especially girls in rural areas. (Malawi poverty reduction strategy p.7). Children enrolled but absent due to sickness may have higher rates of schistosomiasis. In future if localised surveys in areas of low school enrolment are contemplated surveyors should consider including children who are not attending school or are absent due to illness.

Drug treatment has been widely if intermittently available. The National Schistosomiasis Control Programme does not have documented evidence of where and when universal drug treatment has been offered to communities. Nor does it have details of where and when Praziquantel has been available for prescription to symptomatic individuals at health centres and hospitals. Supplies and universal treatment programmes have tended to depend on intermittent interest and funding from NGOs. However Praziquantel seems to have been widely available (Kayuni SA. National Survey to find out difficulties people face in taking regular anti-schistosomiasis drugs in Malawi. Fourth Year Student Project. College of Medicine, Blantyre, 2003) and may have had an effect. In this survey of 264 people living in high risk villages in eleven randomly selected districts 71% reported receiving anti-schistosomiasis treatment.

Several NGOs such as Save the Children (US) in Mangochi and World Vision in Chikwawa in recent years have studied helminth infections in schoolchildren. Save the Children (US) surveyed and treated schoolchildren in the entire Mangochi District after finding a prevalence of *Ascaris lumbricoides *(1.9%), Hookworm (24%), and *S. mansoni *(16%) in four schools. The last survey done in 2000 showed an overall prevalence of *A. lumbricoides *(0.4%) and Hookworm (1.5%) on the four schools after two consecutive courses of treatment (Mary Mukaka, Save the Children, personal communication). Similar interventions may have had an effect on the prevalence of infections in other parts of the country.

Another possible explanation for the unexpectedly low prevalence results could be due to a sampling method that selected a low preponderance of schools in high risk areas. However the results of the analysis looking at expected and actual prevalence found in each school suggest this did not happen. Unexpected results were found in the expected low prevalence as much as in the expected high prevalence schools using the McNemar test for symmetry actual (p = 0.29).

The reason for the comparative low prevalence rate of *S. haematobium *now is not due to asymmetric sampling of low risk schools; it is due to a marked trend of a reduced prevalence across the board. It is not just the high risk schools that have lower prevalence now. Low risk schools have lower rates as well. Compared to the historical results for Malawi districts compiled by the London School of Hygiene and Tropical Medicine for the Schistosomiasis Control Initiative in 2001, prevalence has fallen overall by 24%, and this reduction is true for low as well as high prevalence schools. Our interpretation is that the results reflect a widespread reduction in schistosomiasis prevalence and not a biased sample in the current survey which inadvertently missed schools in high risk areas. However bias may still be an explanation for the change. The widespread reduction in prevalence could be due to bias in the selection of high risk schools in the historic studies rather (than in the selection of low risk schools in our survey). Our sampling fraction of 30 of a possible 3,900 schools is sufficient to give statistical confidence in our overall prevalence result of 6.9% (95% CI 1.9 – 11.9%) for *S. haematobium *so long as our schools and Traditional Authorities were chosen randomly, which they were.

### Questionnaire results

Of the questions asked, only the red urine question is sufficiently sensitive and specific to be used as a screening test. It is a cheap way of assessing schistosomiasis prevalence and as such may be useful to identify hot spots within districts.

### Implications for the control programme

Past control programmes, despite their fragmentary application in Malawi, seem to have achieved much in recent years. High intensity infections are now rare, and it is of course these that are thought to cause morbidity. There remain some high prevalence locations but these are uncommon now. The survey findings suggest that future approaches to control can focus on the identification of hot spots through local surveys. These should be undertaken in districts in high prevalence ecological zones, probably through "red urine" questionnaire surveys of school children. Because of the low positive predictive value of the "red urine" question, a low threshold should be used to categorise each school. The categorisation as proposed in Table 5 takes this into account and provides a simple way to identify those schools that require a urine microscopy survey.

## Conclusions

The highly effective drugs now available and the control methods used in Malawi seem to have reduced the prevalence of both schistosomiasis and soil helminths to low levels.

There remain some localised areas of high prevalence for which local control measures will be required. It seems that the epidemiology of these diseases makes local control at district level or below the preferred public health approach.

## Competing interests

The author(s) declare that they have no competing interests.

## Authors' contributions

BS conceived the survey and provided logistical advice. CB designed the survey and undertook the final analysis and first drafting. BP supervised the survey and undertook the initial analysis. PM undertook the randomization and led the survey team. MP supervised the quality control of the laboratory work. All contributed to the final report.

## Pre-publication history

The pre-publication history for this paper can be accessed here:



## Supplementary Material

Additional File 1 questionnaire; the questionnaire in Chichewa used in the schools to find out about the health of the pupils surveyed. Click here for file

## Figures and Tables

**Table 1:** Prevalence of schistosomiasis and Soil Transmitted Helminths in Malawi primary schools survey, 2002

**Schistosomiasis**	**Prevalence {high intensity} – (95% confidence intervals)**
*S. mansoni*	0.4 (0–1.3) %
S. haematobium*	6.9 (1.9–11.9)% {2.5 (0.2–4.9) %}
Mixed	7.7 (2.2–13.2)%
**Soil transmitted helminthiasis**	
Hookworm	1.3 (0.4–2.3)% {0}
Ascariasis	0.5 (0.1–1.0)% {0}
Trichuriasis	0.0
Any infection	1.8 (0.6–3.1)% {0}

*Heavy intensity *S. haematobium *> or = 50 eggs/ 10 ml or visible haematuria

**Table 2:** Prevalence of helminth and schistosomiasis infections in standard three school children in Malawi, by ecological area.

Nos infected/nos examined [%(range)]	Northern Highland	Central Highland	Central low/lakeshore	South Highland	South low/lakeshore	urban
No. children sampled	**257**	**242**	**291**	**261**	**297**	**277**
**Schistosomiasis**						
*S. mansoni*	0	-	0	7 [1.3(0–4.3)]	0	0
*S. haematobium*	15 [7.4(0–14.8)]	6 [2.0(0–5.0)]	28 [9.5(0.4–18.5)]	14 [3.2(0–8.6)]	66 [23.2(3.3–43.1)]	11 [3.4(0–7.8)]
Mixed	-	-	-	1 [0.2(0–0.6)]	-	-
Any infection	15 [7.4(0–14.8)]	6 [2.0(0–5.0)]	28 [9.5(0.4–18.5)]	20 [4.4(0–11.8)]	66 [23.2(3.3–43.1)]	11 [3.4(0–7.8)]
**Soil transmitted helminthiasis**						
Hookworm	7 [2.6(0.7–4.4)]	4 [1.8(0–3.6)]	0	3 [0.7(0–1.8)]	7 [1.9(0–4.0)]	5 [2.0(0–5.6)]
Ascaris	2 [0.5(0–1.5)]	1 [0.6(0–1.6)]	0	2 [0.4(0–1.1)]	2 [0.3(0–1.2)]	11 [5.1(0.6–9.5)]
Trichuris	-	-	-	-	-	-
Any helminths	9 [3.1 (0–6.4)]	5 [2.4(0–5.2)]	0	5 [1.0(0–2.7)]	8 [2.1(0–4.5)]	15 [6.6(1.3–12.0)]

**Table 3:** Answers to questions about haematuria, blood in stools in the last month and history of bilharzia and worms

**Question**	**Blood in urine**	**Blood in stool**	**Bilharzia**	**Worms**
**No**	1207	1394	1162	1090
**Yes**	384	179	434	512
**Missing answer**	71	89	66	59
**Total**	1662	1662	1662	1661

**Table 4:** Sensitivity and specificity of the "red urine" question

**Answer to**	**Microscopy for *S. haematobium***		**Totals**
		
	**positive**	**negative**	
**Red urine? – yes**	90	289	379
**Red urine? – no**	44	1142	1186
**Totals**	134	1431	1565

**Table 5:** Proposed categorisation of and intervention recommended in schools based on "red urine" prevalence

**Prevalence of positive "red urine" question**	**Intervention recommended**
> 30%	Undertake microscopy survey – if prevalence > 50% – Annual universal treatment. If <50% – Annual targeted treatment to children
< 30%	"Red urine" screen of schoolchildren every two years
