# Supplementary material for: A national survey of the prevalence of schistosomiasis and soil transmitted helminths in Malaŵi
Source: BMC Infect Dis. 2004 Nov 16;4:49. doi: 10.1186/1471-2334-4-49 (PMC535894; doi:10.1186/1471-2334-4-49)
Supplement: Additional File 1 — questionnaire; the questionnaire in Chichewa used in the schools to find out about the health of the pupils surveyed. [file 1471-2334-4-49-S1.rtf]

Questionnaire
HEALTH RESEARCH IN PRIMARY SCHOOLS OF MALAWI
Questions concerning the health of pupils

Explanation: Put a tick () for “yes” or a zero (o) for “no” and a dash (-) if a child does not remember or cannot answer.  You have to answer the following questions.  Each box is for only one child.  If the boxes are not enough on one page, use another sheet. Use only for standard 3. Return this and other sheets to the Headmaster when you finish.  

Name of School: ________________________________	Standard ____________ 	Total number of pupils in class_______________	T/A:  __________________
	
Ana a sukulu																															
Zaka																															
Mwamuna/mkazi (F/M)																															
Mudzi wa mwana wa sukulu																															

Question 1:Kodi ndi ziti mwa zizindikiro izi zomwe zakuchitikira mu mwezi wathawu? Put a tick () or a zero (o) or a dash (-) in the boxes in front of corresponding symptoms

Kutsokomola																															
Kuyabwa																															
Mutu kupweteka																															
Kutentha thupi																															
M´mimba kupweteka																															
Kusaona/khungu																															
Kukodza nkodzo wa magazi																															
Kuchita chimbudzi chamagazi																															

          Question 2: Kodi ndi ati mwamatenda awa omwe wadwalapo pa mwezi wathawu? (Put a tick () or a zero (o) or a dash (-) in the boxes in front of the corresponding diseases)

Malungo																															
Kutsekula mmimba																															
Matenda a pakhungu																															
Matenda a maso																															
Likodzo																															
Matenda a chibayo																															
Njoka za mmimba																															
Kupweteka mmimba																															
Teacher´s  Name: ________________________________	Signature : ____________________________ 	Date: _________________________
